# Network Analysis in Disorders of Consciousness: Four Problems and One Proposed Solution (Exponential Random Graph Models)

**DOI:** 10.3389/fneur.2018.00439

**Published:** 2018-06-12

**Authors:** John Dell'Italia, Micah A. Johnson, Paul M. Vespa, Martin M. Monti

**Affiliations:** ^1^Department of Psychology, University of California, Los Angeles, Los Angeles, CA, United States; ^2^Brain Injury Research Center, Department of Neurosurgery, David Geffen School of Medicine at UCLA, Los Angeles, CA, United States

**Keywords:** network analysis, exponential random graph model, functional magnetic resonance imaging, coma, disorders of consciousness

## Abstract

In recent years, the study of the neural basis of consciousness, particularly in the context of patients recovering from severe brain injury, has greatly benefited from the application of sophisticated network analysis techniques to functional brain data. Yet, current graph theoretic approaches, as employed in the neuroimaging literature, suffer from four important shortcomings. First, they require arbitrary fixing of the number of connections (i.e., density) across networks which are likely to have different “natural” (i.e., stable) density (e.g., patients vs. controls, vegetative state vs. minimally conscious state patients). Second, when describing networks, they do not control for the fact that many characteristics are interrelated, particularly some of the most popular metrics employed (e.g., nodal degree, clustering coefficient)—which can lead to spurious results. Third, in the clinical domain of disorders of consciousness, there currently are no methods for incorporating structural connectivity in the characterization of functional networks which clouds the interpretation of functional differences across groups with different underlying pathology as well as in longitudinal approaches where structural reorganization processes might be operating. Finally, current methods do not allow assessing the dynamics of network change over time. We present a different framework for network analysis, based on Exponential Random Graph Models, which overcomes the above limitations and is thus particularly well suited for clinical populations with disorders of consciousness. We demonstrate this approach in the context of the longitudinal study of recovery from coma. First, our data show that throughout recovery from coma, brain graphs vary in their natural level of connectivity (from 10.4 to 14.5%), which conflicts with the standard approach of imposing arbitrary and equal density thresholds across networks (e.g., time-points, subjects, groups). Second, we show that failure to consider the interrelation between network measures does lead to spurious characterization of both inter- and intra-regional brain connectivity. Finally, we show that Separable Temporal ERGM can be employed to describe network dynamics over time revealing the specific pattern of formation and dissolution of connectivity that accompany recovery from coma.

## 1. Introduction

In the past 15 years, *in vivo* studies of the healthy and diseased brain have increasingly focused on approaches aimed at assessing the spontaneous functional architecture of the brain, conceived as a network of interacting regions (1). Network analyses have been successfully employed in many fields, including sociology (2), computer sciences (3), public health (4), epidemiology (5), and transportation (6), among others, to capture salient aspects of each phenomenon. Indeed, while different fields often employ different approaches to assessing network properties, they all share the common goal of characterizing important aspects of complex network function into a limited number of metrics, which can, jointly, capture both what is unique and what is shared across systems. Network approaches have also been extensively employed toward understanding specific aspects of cognition [e.g., (7)], development (8) and aging (9), and, perhaps most frequently, the pathological brain [e.g., Alzheimer's disease (10), Parkinson disease; (11), severe brain injury; (12)]. This approach has also found fruitful application in the study of human consciousness [e.g., (13–15)]. Indeed, many of the proposals of how human consciousness arises from neural function often make reference to aspects of brain activity as a network of interacting areas, such as the reverberation and spread of neural activity across fronto-parietal association regions (16, 17), the presence of synchronized long-range activity in specific frequency bands [e.g., (18, 19)] and specific neural circuits [e.g., cortico-thalamic loops; (20)], the dynamic competition between assemblies of cells (21), or to the degree to which a network possesses certain topological characteristics [e.g., integration and differentiation; (22)].

In the context of disorders of consciousness [DOC; (23)], network approaches to the study of functional connectivity have given rise to a fertile body of literature (see 24, for a recent review). Yet, there are a number of important methodological challenges which might play into the interpretation of such studies [cf., (25, 26)] and which might explain some of the contrasting results reported [e.g., the exact role of thalamo-cortical vs. cortico-cortical connectivity in recovery of consciousness; see (27–34)]. [See also (35) for further discussion].

In what follows, we propose that it is best to have both seed based and graph theoretic questions in a single model. In the neuroimaging literature, there are a number of limitations of current approaches which have hindered the ability to use a single model for combining seed based and graph theoretic approaches, but there are models that have been developed by other fields (36–40).

### 1.1. Four problems in current network analysis approaches

Current graph theory methods as employed in neuroimaging (41, 42) suffer from a number of important shortcomings which are particularly relevant in the domain of DOC. (We note that the following discussion is in the context of network analysis as currently implemented for neuroimaging data, and is not meant to imply that other fields have not found solutions to them. In fact, as we will argue below, we are advocating for importing into the field of neuroimaging methods that have successfully been applied in other domains).

#### 1.1.1. Problem #1: arbitrary enforcing of network density

Conventional graph theoretic approaches in neuroimaging require sparse networks. That is to say, they require networks (i.e., connectivity matrices) to have some connections (i.e., edges) with non-zero values (typically integer, in binary networks, or fractional, in weighted networks) and some with zero values—as opposed, for example, to fully connected networks in which all edges have non-zero values (i.e., each node is connected to all other nodes with non-zero edges). Yet, since brain networks are typically derived from pairwise correlations across time-series of regions of interest, the starting point for network analysis is typically a fully connected network [in fact, a complex network, which is both fully connected and has positive and negative edges; (43)]. It is thus common procedure to make the connectivity matrices sparse by fixing their density (i.e., the proportion of non-zero edges to the total number of possible edges), which is done by retaining the strongest *d* connections and setting all remaining ones to zero. The resulting network is thus sparse, with density $\frac{d}{N\left(N-1\right)/2}$, where *N* is the number of nodes in the network. On the one hand, this procedure ensures that any uncovered difference across networks (e.g., patients vs volunteers; time-point A vs time-point B) reflects some systematic aspect of their topological characteristics and not, more trivially, the fact that they have different densities. On the other hand, however, because of the lack of a principled approach to perform this procedure, it is currently typical to iteratively re-calculate network characteristics at several density levels, from a lower bound meant to ensure that networks are estimable [such that the average nodal degree is no smaller than 2 × log(*N*); (44)] to an upper bound such that the mean small-world characteristic of networks is no smaller than 1 or 1.5 [e.g., (13)]. While conventional, the idea of enforcing graphs to have the same density across groups, time-points, or conditions is in itself problematic, because it is not hard to imagine that some graphs might be naturally denser than others [see (45)]. This is particularly relevant in the context of the typical comparisons of interest in DOC such as patients vs. healthy volunteers, patients in a Vegetative State vs. patients in a Minimally Conscious State (vs. patients in a Locked-in Syndrome), or within-patient changes over time (e.g., acute-to-chronic designs). Of course, similar problems are encountered in many other contexts (e.g., adolescents vs. older adults) and might even apply to normal, within-group, variability in the healthy brain. Mandating equal density across graphs might obscure important differences across conditions of interest, bias results, and lead to spurious findings.

One solution to the problem of network iterative thresholding is to analyze complex networks [i.e., fully connected and signed matrices; (43, 46, 47)]. Yet, despite this problem having been well documented, as shown in a recent review focused on the use of graph-theoretic approaches in the clinical context, less than 7% of 106 published papers (up to April 2016) employed complex matrices (48). All remaining studies only considered non-negative and/or sparse matrices. In addition, it is important to note two potentially unwanted limitations of using complex matrices. First, the use of complex matrices assumes that the probability of connectivity between two regions is spatially stationary, but it is in fact well known to be inversely related to distance at both the neuronal and region levels [see, (49–51)]. Second, the use of complex matrices affects the formulation of some metrics [e.g., modularity; (43, 46)] because positive and negative edges are treated as separate sparse networks, an issue that is further complicated by the frequent use of mean-centering preprocessing strategies which are known to shift the distribution of positive and negative edges (52, 53). Furthermore, the formulation and interpretation of other metrics [e.g., path based metrics such as characteristic path length/local efficiency, betweenness centrality, etc.; (46, 54)], are also affected since the weights represent both the strength and probability of the connections (i.e., density). Thus, analyzing fully connected signed graphs does avoid the thresholding issue but at the cost of clouding the interpretation of metrics such as density and path-based graph statistics.

#### 1.1.2. Problem #2: network measures are not independent of each-other

A standard network analysis, as currently implemented in the field, typically assesses a number of different topological measures in parallel, such as characteristic path length, average clustering, efficiency, and small-world characteristic, among others [c.f., (43)]. Many of these characteristics, however, are not independent of each other. In fact, they are often interrelated and can greatly influence each other (55–57). Consider two metrics often employed in graph theoretic analysis of brain data: clustering coefficient and density. Clustering coefficient can be described as the level of segregated neural processing within a network (42). Density, as explained above, is a measure of the number of existing edges within a network (i.e., connection with non-zero value), divided by the total number of possible edges. These two network characteristics are strongly interrelated: It has been shown that there is a clear relationship between a network's density and its clustering coefficient (57). Similarly, dependencies between many other network measures frequently employed in the neuroimaging literature (e.g., degree, clustering coefficient, characteristic path length, and small world index) have also been reported (55, 56), highlighting the need to control for these relationships in order to minimize the potential for spurious findings [see (42, 55)]. Conventionally, this problem is addressed by arbitrarily fixing network density (see Problem #1). This approach, however, suffers from two important shortcomings. First, as explained above, different networks might well have different levels of natural—or stable—density. Second, it is a rather weak control. For example, it only addresses the dependencies of network measures on density, but ignores the many other known correlations among features of networks that are often assessed [cf., (55)], which, to date, have gone unaccounted for in virtually all of the extant literature in the field.

#### 1.1.3. Problem #3: failure to account for structural information in shaping functional networks

In the clinical context of DOC, despite the fact that patients are well known to have heterogeneous underlying pathology, which introduces many concerns for proper diagnosis (58, 59), functional [e.g., (13, 15, 28, 34, 60–63)] and structural connectivity (64, 65–69) are typically investigated separately. This narrow approach is very problematic because it has been shown, in the rodent model (70) and in healthy humans (71, 72), that structural data can predict the functional connectivity as estimated by correlations in the fMRI signal, as well as EEG phase coupling in healthy volunteers (73). Failing to include both structural and functional data will have a similar effect on the analysis of functional networks as omitting any other graph metric (i.e., problem #2): it will result in improper estimation of the terms in the model and potentially spurious results. This issue is particularly important in the clinical context of DOC given their highly heterogeneous pathology and the fact that this can change over time, which affects longitudinal comparison of brain networks over time.

Diffusion weighted imaging (DWI) and blood oxygenation level dependent (BOLD) can be used in conjunction to estimate connectivity matrices using joint independent component analysis [jICA; (74)], Connectivity Independent Component Analysis [connICA; (75)] or partial least squares [PLS; (76)]. In general, all three methods produce multiple group connectivity matrices based on the covariance of BOLD and DWI data across all participants. Both jICA and connICA produce multiple components that are maximally spatially independent [for a complete explanation of jICA see (77–79) and for a complete explanation of connICA see (33)]. PLS produce a linear combination of latent variables that maximally covary with each other based on weighted structural and functional connections [for a complete explanation of PLS see (80–83)]. These methods incorporate both structural and functional connectivity in the estimation of the connectivity matrices, but they require researchers to choose the number of components (in jICA and connICA) or number of latent variables (in PLS). Changing these parameters influences the results of the connectivity estimation and the standards for these parameters are still being investigated for both jICA and connICA (78, 84–86). We thus propose an alternative to these methods that avoids the necessity to estimate the functional and structural connectivity jointly. In the approach we describe below, the structural and functional connectivity matrices are estimated separately, and the former is used as a variable in estimating graph statistics for the latter (see section 2.6 for a complete description).

#### 1.1.4. Problem #4: network dynamics—estimating network change over time

Finally, contrary to the assumption underlying conventional network analysis in neuroimaging, connectivity between areas is unlikely to be stationary processes. Rather, brain activity might best be viewed as a malleable and variable process over time (87). Yet, even in the few cases where this limitation has been addressed [e.g., (88)], these types of approaches do not quantify dynamic change of connectivity across time (or states). Rather, they just dissect a time-series into multiple static networks and compare them over their respective topological properties. In other words, even these approaches are static in nature and fail to capture the dynamics of network connectivity over time. In the context of DOC, for example, this means that longitudinal analysis of brain data can be employed to reveal differences in topological properties of networks at two different time-points, but do not allow saying anything of the process of interest, which is the dynamics of how one network transitioned into another (e.g., how a network transformed as consciousness was regained over time).

### 1.2. Exponential random graph models

In response to these four shortcomings of current network analysis, we present and demonstrate a novel [in the context of DOC, for other contexts within neuroimaging, cf.: (89–91)] approach to graph analysis, referred to as Exponential Random Graph Models [ERGM; (36)]. The core idea underlying ERGM is that instead of considering graphs as fixed entities which can be described in terms of topological properties (e.g., clustering, path length, small world property), it attempts to generate hypotheses about the (unobserved) stochastic processes that gave rise to an observed network (92). Contrary to the prevalent approach in neuroimaging, then, the presence/absence of an edge within a network is not considered to be a fixed property of a graph, but rather a random variable generated by a stochastic process. In other words, rather than assuming the observed network as “given" and fix, and describing its topological characteristics (e.g., characteristic path length, clustering coefficient), it tries to characterize the processes that have generated the observed network. One particularly appealing aspect of this approach is that, so long as the total number of nodes (i.e., ROIs) constituting a network remains unchanged, it allows for comparing across networks with different density levels, thereby solving problem #1. The ERGM framework uses the following exponential model:

(1) $$\begin{array}{l} P_{\theta }\left(Y=y\right)=\frac{\exp\left(\theta^{T}g\left(y\right)\right)}{c\left(\theta \right)} \end{array}$$

where θ is a parameter vector that is modeled by g(y) (i.e., any statistic of the graph). The parameter *c*(θ) is a normalizing constant representing the parameter estimate for all possible graphs (38). This normalizing constant is not able to be analytically solved due to the combinatorics of the graph structure. We can nonetheless approximate the unknown population mean using *c*(θ_*s*_) (i.e., the sample mean):

(2) $$\begin{array}{l} \frac{c\left(\theta \right)}{c\left(\theta_{s}\right)}=E_{\theta_{s}}\exp{\left(\theta -\theta_{s}\right)}^{T}g\left(y_{i}\right)\\ \frac{c\left(\theta \right)}{c\left(\theta_{s}\right)}\approx \frac{1}{M}\overset{M}{\underset{i=1}{\sum }}\exp{\left(\theta -\theta_{s}\right)}^{T}g\left(y_{i}\right) \end{array}$$

for derivations [see (38)]. These equations allows for an approximation of the population mean using sample mean. A bootstrapping method using Markov Chain Monte Carlo (MCMC) methods is used to sample and estimate the population mean. These methods assume Markovian principles of independent draws and the ability to reach equilibrium. Equilibrium is the state in which any edge that is toggled on or off results in an equally probable graph. The general method is to take the ratio of the probabilities of *Y*_*ij*_ = 1 (i.e., adding a single edge) and *Y*_*ij*_ = 0 (i.e., no edge) conditioned on $Y_{ij}^{C}=y_{ij}^{C}$ (i.e., all other pair of nodes in the graph).

(3) $$\begin{array}{l} \frac{P\left(Y_{ij}=1|Y_{ij}^{C}=y_{ij}^{C}\right)}{P\left(Y_{ij}=0|Y_{ij}^{C}=y_{ij}^{C}\right)}=\exp\theta^{*}\left(s\left(Y_{ij}=1\right)-s\left(Y_{ij}=0\right)\right)\\ \log\frac{P\left(Y_{ij}=1|Y_{ij}^{C}=y_{ij}^{C}\right)}{P\left(Y_{ij}=0|Y_{ij}^{C}=y_{ij}^{C}\right)}=\theta^{*}\Delta \left(s\left(Y_{ij}\right)\right)\\ \text{LPL}\left(\theta \right)=\sum \log\left[P\left(Y_{ij}=y_{ij}\right)|\left(Y_{ij}^{C}=y_{ij}^{C}\right)\right] \end{array}$$

where the LPL(θ) is the log-pseudolikelihood for θ, which is maximized by taking the maximum pseudolikelihood for θ (38). This estimation process is performed for the model with all the parameters (i.e., θ). The estimates give the mean and standard error. These estimates were tested for significance in each functional data set. Due to the MCMC, a t-statistic can be estimated and is reported in the model output along with a p-value.

For interpretation purposes, Equation 1 can be represented as follows [the full derivations can be found in (38)]:

(4) $$\begin{array}{l} \text{logit}\left(P_{\theta }\left(Y_{ij}=1|nactors,Y_{ij}^{C}\right)\right)=\overset{K}{\underset{k=1}{\sum }}\theta_{k}\delta_{Z_{k}\left(y\right)} \end{array}$$

where *k* is the number of network statistics in the model and θ_*k*_ is the parameter estimate for each statistic. The δ_*Z*_*k*_(*y*)_ is the change in network statistic if a edge were added between any node *i* and *j*. Thus, the interpretation of the network statistics involve the change in probability of an adding a edge with certain network statistic. The significance of a parameter estimate is one compared to the expected parameter estimate in a null model with the probability of all edges equal to 0.5 [i.e., (93)].

In what follows, we first demonstrate the insidiousness of problem #2 in the context of well characterized, freely-available, data on the business ties of Florentine families in the fifteenth century (94), and then we apply the powerful and flexible ERGM approach to estimating network statistics for characterizing (brain) networks in the longitudinal context of a patient recovering after coma after severe traumatic brain injury (TBI). To anticipate the key points that will follow, ERGM, which has been successfully employed in other contexts (36–40), offers a number of substantial advantages which are particularly important in the clinical context of DOC. First, it does not require imposing (and assuming) the same level of density across graphs, thus allowing estimating characteristics of each graph at its “natural” density level. Second, it allows for controlling the dependencies between network characteristics. In this sense, in contrast to the conventional approach, which can be viewed as a series of univariate regressions (i.e., one per metric) assessing the topological characteristics across groups of graphs (e.g., patient groups and controls vs. patients), ERGM is making use of a multiple regression framework (39), in which all features are considered together, and thus returns the “unique” contribution of each network measure. Third, the multiple regression framework extends to graph theoretic measures characterizing the structural connectivity of a network, thus accounting and “parceling out” the effect of cross-sectional differences [e.g., (69)] and longitudinal changes in structural connectivity [e.g., (95, 96)] across graphs. Finally, a temporal implementation of this technique, Separable Temporal ERGM (STERGM), allows assessing the dynamic changes of network properties occurring over observations (e.g., time, clinical groups).

## 2. Methods

### 2.1. Florentine business ties data

**Figure 1:**
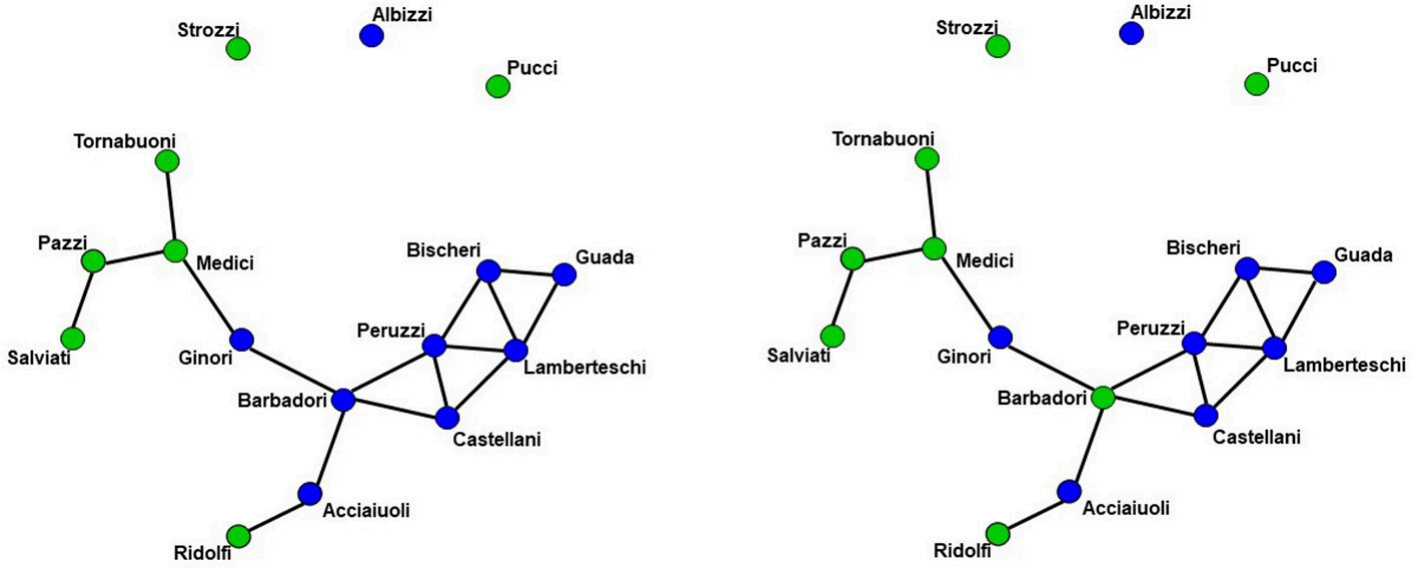
Florentine business ties networks. Florentine business ties data with additional grouping. **Left:** Network A. **Right:** Network B. We note that two networks are identical except for the Barbadori family being allocated to the blue group in the left graph and to the green group in the right graph.

We demonstrate the importance of problem #2 using freely available data for social network analysis. The dataset, which has been extensively characterized in previous work, describes business connections between Florentine families in the fifteenth century (94). We use this data analysis to demonstrate the interrelationship between network measures and how failure to include them in a single full model (FM) can lead to spurious results. Specifically, the relationship between network measures is manipulated by constructing two identical networks with one unique difference between them—that is, whether the Barbadori family belongs to the blue group (Figure 1, left) or the green group (Figure 1, right). As we will discuss further below, this example focuses on the relationship between node mixing terms (i.e., a measure of within-group [blue vs. green] connectivity) and a higher order term called geometrically weighted edge shared partners (GWESP; a type of triangles term; see section 2.6 for full description of both terms). To demonstrate the effects of relationships between measures, we estimate three models per each network: two partial models (PMs) including an edges term and either the higher order term (PM_*A*_) or the mixing terms (PM_*B*_), and the FM containing all terms. As we will show, for each network, PMs return spurious results with respect to both significance and magnitude of the parameter estimates.

### 2.2. Patient

We demonstrate the use of ERGM models using longitudinal data from a patient recovering from a severe brain injury. A 40 to 45 year old person suffered a severe TBI due to a fall. The patient suffered pulmonary contusion and liver laceration, and presented with a post-resuscitation Glasgow Coma Scale [GCS; (97)] of 3. Computerized tomography (CT) revealed skull fractures, traumatic subarachnoid hemorrhage, extradural hematoma, subdural hematoma, and bilateral frontal lobe contusions. At the 3 acute imaging sessions, which occurred on the 11, 18, and 25*th* days post-injury, the patient presented a total GCS of 6 (Eyes opening (E): 1, Verbal response (V): 1, Motor response (M): 4), 7 (E:1, V:1, M:5), and 10 (E:3, V:1, M:6), respectively. While DoC diagnoses are typically not made at such an acute stage, the behavioral profile of the patient was consistent with a vegetative state [VS; i.e., wakefulness in the absence of any behavioral sign of awareness of the self or the environment; (23)] at the first time-point, with a minimally conscious state *minus* [MCS-; i.e., wakefulness with intermittent but reproducible signs of low-level non-reflexive behaviors, such as orientation to noxious stimuli; (98)] at the second time-point, and a an minimally conscious state *plus* [MCS+; i.e., wakefulness with intermittent but reproducible signs of high-level non-reflexive behaviors, such as response to command, intelligible verbalization, or gestural or verbal yes/no responses; (98)] at the third. At 6-months follow-up the patient was assessed with a Glasgow Outcome Scale—Extended [GOS-E; (99)] in-person interview and scored as being in a lower moderate disability (i.e., GOS-E = 5).

### 2.3. Experimental design

The patient underwent 4 imaging sessions over the span of 6 months. The first 3 sessions occurred within a month post injury (see above), and the follow-up session took place 181 days post-injury. At each session the patient underwent (among other clinical and research sequences) anatomical (T1-weighted) and functional (T2^*^-weighted) data protocols. T1-weighted images were acquired with a 3D MPRAGE sequence (repetition time [TR] = 1,900 ms, echo time [TE] = 3.43, 1 × 1 × 1 mm). BOLD functional data were acquired with a gradient-echo echo planar image (TR = 2,000 ms; TE = 25 ms, 3.5 × 3.5 × 4 mm). Diffusion Weighted data were acquired with an echo planar sequence (TR = 9,000 ms, TE = 90 ms, 64 directions, 3 × 3 × 3) using a b-value of 1,000 and acquiring an additional B0 image. Acute data were acquired on the in-patient 3 Tesla Siemens TimTrio system at the Ronald Reagan University Medical Center, while chronic data were acquired on the out-patient 3 Tesla Siemens Prisma system also at the Ronald Reagan Medical Center at the University of California Los Angeles. The study was approved by the UCLA institutional review board (IRB). Informed consent was obtained from the legal surrogate, as per state regulations.

### 2.4. Data preprocessing

#### 2.4.1. BOLD data preprocessing

The functional data underwent a number of conventional preprocessing steps including brain extraction, slice timing correction, motion correction, band-pass filtering (0.08 ≤ Hz ≤ 0.1), and removal of linear and quadratic trends. A nuisance regression was employed to parcel out signals of non-interest including motion parameters, white matter, cerebral spinal fluid, and full-brain mean signal [which has been shown to alleviate the consequences of in-scanner motion; (100)]. Affine registration of the functional data to the standard template (MNI) was performed using Advanced Normalization Tools [ANTs; (101, 102)].

#### 2.4.2. DWI data preprocessing

The diffusion data were preprocessed using the following pipeline: DWI preprocessing, registrations, probabilistic tractography with tractography thresholding. All of these processes were run using a bash script in parallel using the GNU Parallel package (103).

##### 2.4.2.1. DWI preprocessing

All preprocessing procedures were visually checked for optimal quality. The T1-weighted data were brain extracted [optiBET; (104)] and bias field corrected [BrainSuite BFC; (105)]. The diffusion-weighted data were prepared for tractography with the following steps: (1) visual quality checking of raw images; (2) artifact checking/removal and motion correction with vector rotation [DTIprep; (106)]; (3) eddy current distortion correction followed by tensor fitting (i.e., linear fitting using weighted least squares) and estimation of diffusivity metrics [BrainSuite's BDP; (107, 108)]; (4) brain extraction of the b0 image [BET; (109)]; and (5) GPU-enhanced Bayesian estimation of the diffusion profile with up to two principal directions per voxel (i.e., allowing for crossing/kissing streamlines) using FSL's bedpostx (110, 111).

##### 2.4.2.2. Registrations

All registrations were visually checked for optimal quality. The following steps were conducted: (1) linear registration of the native diffusion data (b0 image) to the native T1-weighted data [ANTs IntermodalityIntrasubject; (102)]; (2) nonlinear registration (ANTs) of the native T1-weighted data to the Montreal Neurological Institute (MNI) standard space (MNI Avg 152 T1 2 × 2 × 2 mm standard brain); (3) forward or inverse transform concatenations [ANTs; (102)] to move between native diffusion, native T1, and the MNI template.

##### 2.4.2.3. Probabilistic tractography

GPU-enhanced probabilistic tractography between all regions of the whole-brain atlas (i.e., iteratively seeding from each region to all other regions as targets) was conducted with the “matrix1” option in FSL's probtrackx2 (110, 112). A minimum distance of 4.8 mm (i.e., 2 voxel widths) was set to prevent artificial streamlines passing through contiguous regions. The output matrix of streamline counts between all regions was thresholded to remove spurious streamlines with an optimization procedure that minimizes asymmetries between the seed/target assignments for each ROI-ROI pair [MANIA; (113)].

### 2.5. Brain network construction

**Figure 2:**
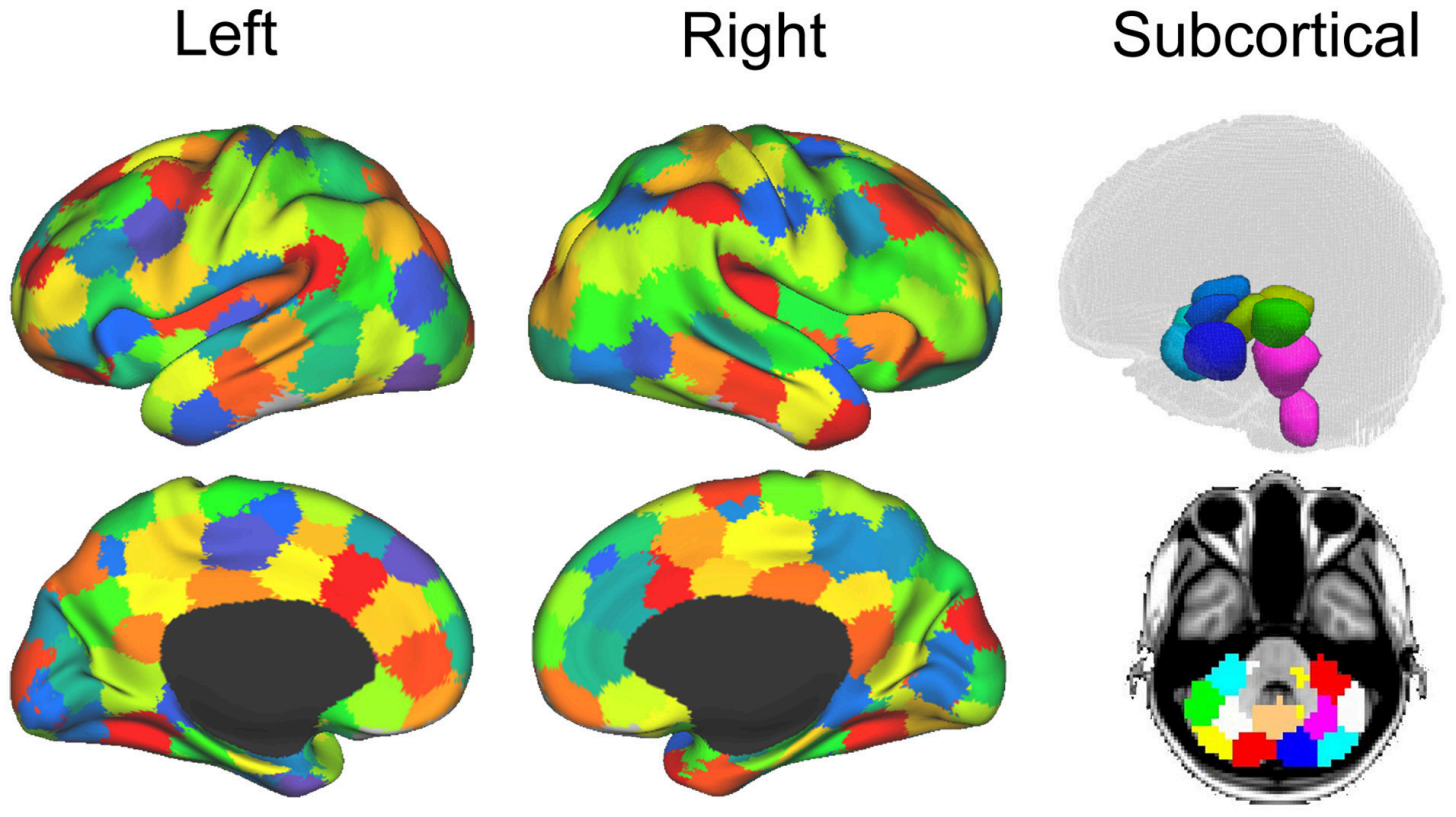
Parcellation for structural and functional connectivity. Cortical and subcortical parcellation of the brain data (114). The imaging sessions' data sets were parcellated into 148 ROIs throughout the cortex, sub-cortical nuclei, cerebellum and brainstem. Figure from (13).

For each dataset (both the functional and diffusion data), a graph was constructed to provide a mathematical description of the brain as a functional network. Brain graphs were constructed in two steps. First, these data sets were parceled into 148 ROIs spanning the cortex, sub-cortical nuclei, cerebellum and brainstem (see Figure 2). This parcellation scheme, which was defined independently of our data, is made freely available by Craddock and colleagues (114). Additionally, we used the Oxford thalamic connectivity atlas (115) to further refine the parcellation of the thalamus from 6 to 14 for a total of 148 ROI (i.e., 134 Craddock ROIs and 14 Thalamic ROIs). While other parcellation schemes are available (e.g., Harvard-Oxford atlas, AAL atlas), the present one has two main advantages [cf., (13)]. First, being functionally defined, it clusters spatially proximal voxels by the homogeneity of their functional connections as opposed to clustering voxels by anatomical position which, as exemplified by the case of the precentral gyrus ROIs in both the AAL and the Harvard-Oxford atlases, might cluster together functionally distinct sub-regions. Second, at our chosen level of resolution, the Craddock ROIs have almost 50% more granularity as either structural atlas (i.e., 148 ROIs vs., 90 and 112 for the AAL and Harvard-Oxford atlases, respectively). Following parcellation, the average time-course of all voxel within each ROI were extracted and correlated across each pair of regions.

Functional connectivity was assessed with a partial correlation method using the Markov Network Toolbox [MoNeT; (116)] in MATLAB. This approach, referred to as R3 (as in resampling, random penalization, and random effects), combines a penalized maximum likelihood estimation—or graphical lasso—procedure with a resampling-based (bootstrapped) model selection procedure, on whitened BOLD timeseries, to infer fully-data driven stable functional connectivity estimates at the single-subject (or group) level. Under this approach, each fMRI time series is repeatedly bootstrapped in order to estimate the within-subject variability and matrices of penalty parameters which reduce selection bias and variability. This method thus reduces the spurious connections from indirect sources arising from the high dimensionality of fMRI data often seen when using the conventional Pearson's r method. Using partial correlations with regularization parameters, the indirect sources are eliminated and the sparsity of each matrix is determined by the within subject variability. Thus, each functional data set returns a connectivity matrix that represents connectivity from direct sources, rather than indirect ones, and that is sparse, as determined on a single-subject basis through bootstrapping and regulatization. This latter point side-steps entirely the need for arbitrary and iterative thresholding approaches (42). It is important to point out, however, another important difference between the partial correlations approach described above and the standard correlation approach to estimating brain networks as performed by most previous work [e.g., (13, 117, 118)]. On the one hand, the conventional correlational approach has the advantage of allowing straightforward interpretation of the elements of adjacency matrices as strength of the functional connectivity between nodes. On the other hand, the matrices generated are fully connected and thus requiring application of a non-linear transformation (e.g., thresholding) in order to render them sparse – a condition necessary for application of many common graph theory metrics (42). In contrast, the partial correlation method employed here returns a sparse matrix. However, it does so at the cost of losing interpretability of graph weights which can now be seen as the functional connectivity between two nodes *i* and *j* after controlling for the correlations with other nodes in the neighborhood (i.e., connected with) – say – *i*. For this reason, matrices obtained with this novel methodology are typically binarized, thus resulting in a sparse matrix of ones and zeros indexing the presence/absence of functional connectivity between each pair of nodes (i.e., ROIs).

### 2.6. Graph statistics

All ERGM models we used to analyze the patient data included the same graph statistics. The model used for all the data sets was specified as follows:

(5) $$\begin{array}{l} P_{\theta }(Y=y)=\frac{\begin{array}{l} \text{exp}(\theta_{1}\text{edges}+\theta_{2}\text{nodecov}(\text{degree})+\theta_{3}\text{nodecov}(\text{efficency})+\theta_{4}\text{nodecov}(\text{cluster})+\theta_{5}\text{nodemix}(\text{latent})\\ +\theta_{6}\text{nodemix}(\text{resting})+\theta_{7}\text{gwesp}(\text{alpha}=\lambda )) \end{array}}{c(\theta )} \end{array}$$

Edges refers to the total number of edges for each functional connectivity graph. This term allows control for the density of each graph. In this sense it is thus similar to the intercept in a linear regression and is thus typically not interpreted or further analyzed.

There are four nodal covariate terms for the diffusion data—three nodal covariates (i.e., degree, efficiency and cluster) and the nodemix (latent) term –and a nodal covariate for the functional connectivity (i.e., nodemix for resting). Degree is the number of edges for each structural node. Efficiency is the local efficiency of each node. Cluster is the clustering coefficient of each node. The nodecov term estimates the probability of functional connectivity edge as a function of each distribution of the structural terms (i.e., degree, local efficiency and clustering coefficient). A positive coefficient indicates an increase in the probability of a functional connectivity edge as structural term increases in magnitude. On the other hand, a negative coefficient indicates an increase in probability of a functional connectivity edge as the structural term decreases.

As shown in Equation (5), there are two nodemix terms: latent and resting. The nodemix (latent) is the within and between module connectivity of the structural connectivity. Thus, this mixing term represents the probability of a functional connectivity edge given the modular membership based on the structural connectivity. The number of modules and modular membership of each node is determined by a position latent cluster ERGM (119, 120). These models have shown to be able to use a latent space model with an a priori determined number of dimensions using the parameter d (3 dimensions). The nodes are arranged in a euclidean system with proximity equating to probability of an edge. The clusters are determined by the parameter G (3, 4, 7, and 6 for Acute first, second, third sessions and Chronic session, respectively). This parameter sets the number of Gaussian spherical clusters that are introduced in the latent space. The estimation of position latent cluster ERGM is a two step Bayesian estimation, but the exact specification is beyond the scope of this paper [see (119)].

The nodemix (resting) is our mixing term for determining the inter- and intra-regional connectivity of the resting state networks and sub-cortical regions of the functional data. Multiple parameter estimates were produced for this term. Additionally, these mixing terms used the exogenous node labels for each node's membership in the seven resting state networks (121) and sub-cortical regions. Each node of the brain network was labeled either: frontoparietal, visual, somato-motor, limbic, dorsal attention, ventral attention, default, subcortex and thalamus. Each combination of the inter- and intra-regional connectivity produced a mixing term and parameter estimate. For example, one inter-regional mixing term would be frontoparietal and thalamic connectivity. This parameter estimate would give the probability of an edge existing between the frontoparietal network and thalamus. An example of intra-regional mixing term would be frontoparietal to frontroparietal. This term would express the probably of an edge within the frontoparietal network. These mixing terms were used to assess the connectivity between the within the resting state networks, between the resting state networks, within the sub-cortical regions, between the sub-cortical regions, and between resting state networks and sub-cortical regions. This term incorporates questions that would be addressed using seed based connectivity analyses.

The geometrically weighted edged shared partners (GWESP) can be expressed by this equation (37):

(6) $$\begin{array}{l} \theta_{t}=log\lambda_{t}\\ v\left(y;\theta_{t}\right)=e^{\theta_{t}}\overset{n-2}{\underset{i=1}{\sum }}\left[1-{\left(1-e^{-\theta_{t}}\right)}^{i}\right]EP_{i}\left(y\right) \end{array}$$

In this equation, *v* is the GWESP term and θ_*t*_ is the log of the decay parameter that was fixed in all the data sets. The *EP*_*i*_(*y*) is the edge shared partners term for the entire graph. It accounts for the number of each type of edge shared partner. An edged shared partner is triangle that shares a common base. Edge shared partners is a metric used to quantify the amount of clustering in the form of transitivity in a network. High positive parameter estimates indicate that transitivity is present above and beyond all the other statistics in the model. Transitivity is a higher order relationship present in most graphs which are the local and/or global communication and the amount of local cohesion. Differences in transitivity between patients could be a key change that occurs from injury. This would be a disruption of the clustering found within the patient's brain. This type of disruption would hamper local and/or global communication and additionally it would indicate a lack of local cohesion within a network.

The analysis was performed using the ERGM package (40) in R. There are two ERGMs used on the patient data. A FM and used all the terms from Equation (5). The FM was fit multiple times to get assess the proper λ (the decay parameter) for the GWESP term. The range of λ began at 0.05 and increase by increments of 0.05 up to 2.0. Each iteration was checked by inspecting the diagnostics of the MCMC. The models that have the best fit for the parameter estimate GWESP were chosen (i.e., λ = 0.45). A second model, the PM was fit. The structural terms (i.e., the three nodecov and the nodemix for latent) were omitted from this model to demonstrate the effects on the rest of the parameter estimates.

The FM's graph statistics were chosen based on two reasons: the type of functional data being analyzed (i.e., resting state data) and the first three problems outlined above (see sections 1.1.1–1.1.3). The nodemix (resting) terms were chosen because this patient's functional connectivity matrices were estimated from the BOLD correlations during the resting state scans. Thus, the intra- and inter- regional connectivity would be best characterized by putative resting state networks. The number of resting networks were chosen based on a data driven approach [i.e., (121)] that estimates a number of networks based on stability of clusters [for details on the clustering algorithm see (122)] estimated from 1,000 subjects' functional data. A seven network parcellation was chosen because it minimized the instability (121) and matches what has been previously discussed in the literature [e.g., (123–126)]. Additionally, the thalamus group was added because of its possible involvement in DOC [e.g., (28, 29, 32, 127)] or anesthesia induced loss of consciousness [e.g., (117, 118, 128, 129)]. Finally, the subcortical and cerebellum groups were added to ensure every node fit a grouping label.

The edges term allows for networks with varying density to be modeled and compared (cf., Problem #1, section 1.1.1). The higher order term (i.e., GWESP) describes the local and/or global communication which could be an important aspect in the recovery from brain injury [e.g., (14, 32, 130)], and because it alleviates the problem of interrelation among graph theoretic measures (cf., Problem #2, section 1.1.2) by accounting for the higher order term's variance and thus avoiding it being improperly allocated to lower order terms (i.e., edges, node mixing, and structural terms). As shown below, failing to include the higher order term can affect the estimation of parameters in either magnitude or sign. Structural connectivity is important because, as stated in third problem (cf., section 1.1.3), it can be severely affected by TBI, systematically changing over time and/or patient cohorts, and because it is interrelated with functional connectivity. Thus, we chose four terms for the structural connectivity that would capture the number of connections of each node (i.e., degree), a measure of integration [i.e., local efficiency (42), and higher order relationships (i.e., clustering and modularity). The two higher order terms were chosen because they capture two different higher order dynamics: local grouping of nodes [i.e., clustering coefficient (42)] and community structure [i.e., modularity; (42)].

The models were assessed by using goodness of fit (GOF) plots (38). After the model was estimated, a thousand simulations were run from the model statistics. These simulations were compared to the original graph's probabilities for each graph statistic (e.g., the probability of nodes with a specific degree, probability edge shared partners and the probability minimum geodesic distances). This is to ensure that the model represents a graph similar to the original data that it was modeled from. The metrics chosen for this example is degree distribution, edge wise shared partner, minimum geodesic distance (another form of local path length) and the nodal covariates from Equation 5. These are the most commonly used graph metrics because they capture important characteristics of graphs that capture the central tendencies and clustering of graphs. The MCMC diagnostics were assessed for each parameter estimate. The GOF plots were used to assess the fit of the FM and all four GOF plots was assessed for goodness of fit.

### 2.7. Separable temporal exponential random graph model

STERGM (131) is an extension of the original ERGM. It is used to assess the dynamics of networks as they change over time. The same underlying methods for estimating ERGM is used in STERGM. A model with network statistics is used to estimate the parameter estimates for a network that changes over time. To achieve this, two separate networks are investigated. A formation network is generated conditional on forming edges,

(7) $$\begin{array}{l} P\left(Y^{+}=y^{+}|Y^{t};\theta^{+}\right)=\frac{exp\left(\theta^{+}g\left(y^{+},X\right)\right)}{c\left(\theta^{+},X,Y^{+}\left(Y^{t}\right)\right)},y^{+}\in Y^{+}\left(y^{t}\right) \end{array}$$

where a formation network *Y*^+^ is characterized by formation parameters θ^+^ (131). The formation network statistics are *g*(*y*^+^, *X*) and the normalizing constant is *c*(θ^+^, *X, Y*^+^(*Y*^*t*^)). The second network formed is a dissolution network that is conditional on the edges that dissolve. This network is represented by the same variables labeled with minus instead of a plus,

(8) $$\begin{array}{l} P\left(Y^{-}=y^{-}|Y^{t};\theta^{-}\right)=\frac{exp\left(\theta^{-}g\left(y^{-},X\right)\right)}{c\left(\theta^{-},X,Y^{-}\left(Y^{t}\right)\right)},y^{-}\in Y^{-}\left(y^{t}\right) \end{array}$$

where a dissolution network *Y*− is characterized by dissolution parameters θ− (131). The dissolution network statistics are *g*(*y*−, *X*) and the normalizing constant is *c*(θ−, *X, Y*−(*Y*^*t*^)). These networks can form a new network at time *t*+1 by applying formation and dissolution networks on *y*^*t*^. This can be expressed as:

(9) $$\begin{array}{l} Y^{t+1}=Y^{t}\cup \left(Y^{+}-Y^{t}\right)-\left(Y^{t}Y^{-}\right) \end{array}$$

The formation and dissolution networks are independent of each other across the *t*+1 time points (131). STERGM has the unique ability to model networks as they transform over time enabling research questions about the dynamics of a network. The same model in Equation 5 was used in both the formation and dissolution models. The quantifications of these networks are similar to ERGM, but these two models slightly change the interpretation of the parameter estimates. In the formation model, a positive parameter estimate indicates a tendency for edges for a network statistic form at time point *t*+1, and a negative parameter estimate indicates a lack of formation of edges for a particular network statistic at time point *t*+1. The dissolution model has two separate interpretations based on the sign of the parameter estimate. A negative parameter estimates are interpreted as edges are more likely to dissolve and positive parameters indicate edges are more likely to be preserved. Despite these differences in interpretation, all the same procedures were used in STERGM as were used in ERGM (PM, FM, quality control using MCMC diagnostics, and assessing fit using GOF) for both the formation and dissolution models.

## 3. Results

### 3.1. Florentine business ties

Network A has both the mixing term and triangles term as significant model statistics when modeling them separately (i.e., PM_*A*_ and PM_*B*_ see Table 1). When they are combined together into the FM, the mixing term remains significant but the triangle term is no longer significant. Thus, the FM for the Florentine business ties properly attributes the variance of each graph theory statistic and the selective mixing term remains significant. The network B has just the triangles term significant in the PM_*A*_ and FM. The mixing term is neither significant in the PM_*B*_ nor the FM.

**Table 1:** Florentine business ties models.

	**ERGM Parameter Estimates**					
	**Network A**			**Network B**		
	**PM**_*A*_	**PM**_*B*_	**FM**	**PM**_*A*_	**PM**_*B*_	**FM**
Edges	−2.44^***^	−3.42^***^	−3.54^***^	−2.46^***^	−2.27^***^	−2.75^***^
	(0.40)	(0.72)	(0.70)	(0.39)	(0.43)	(0.49)
Nodal Covariate Mixing: Within Group 0		1.63	1.60		0.15	0.31
		(0.95)	(0.88)		(0.75)	(0.65)
Nodal Covariate Mixing: Within Group 1		2.60^**^	2.16^**^		1.17	0.91
		(0.80)	(0.82)		(0.61)	(0.48)
GWESP (Fixed 0.8)	0.53^*^		0.32	0.54^*^		0.50^*^
	(0.23)		(0.28)	(0.23)		(0.23)

*Three models are run on each network in Figure 4: PM_A_, PM_B_, FM. The PM_A_ has just the edges and triangles term. The PM_B_ has just the edges and mixing term. The FM has all three terms. Each term has a parameter estimate, a standard error in parenthesis and a p-value indicated by asterisks. The LATEX code to create this table was produced by the R package called texreg (132)*.

* *p < 0.05*;

** *p < 0.01*;

*** *p < 0.001*.

### 3.2. Patient recovery

**Figure 3:**
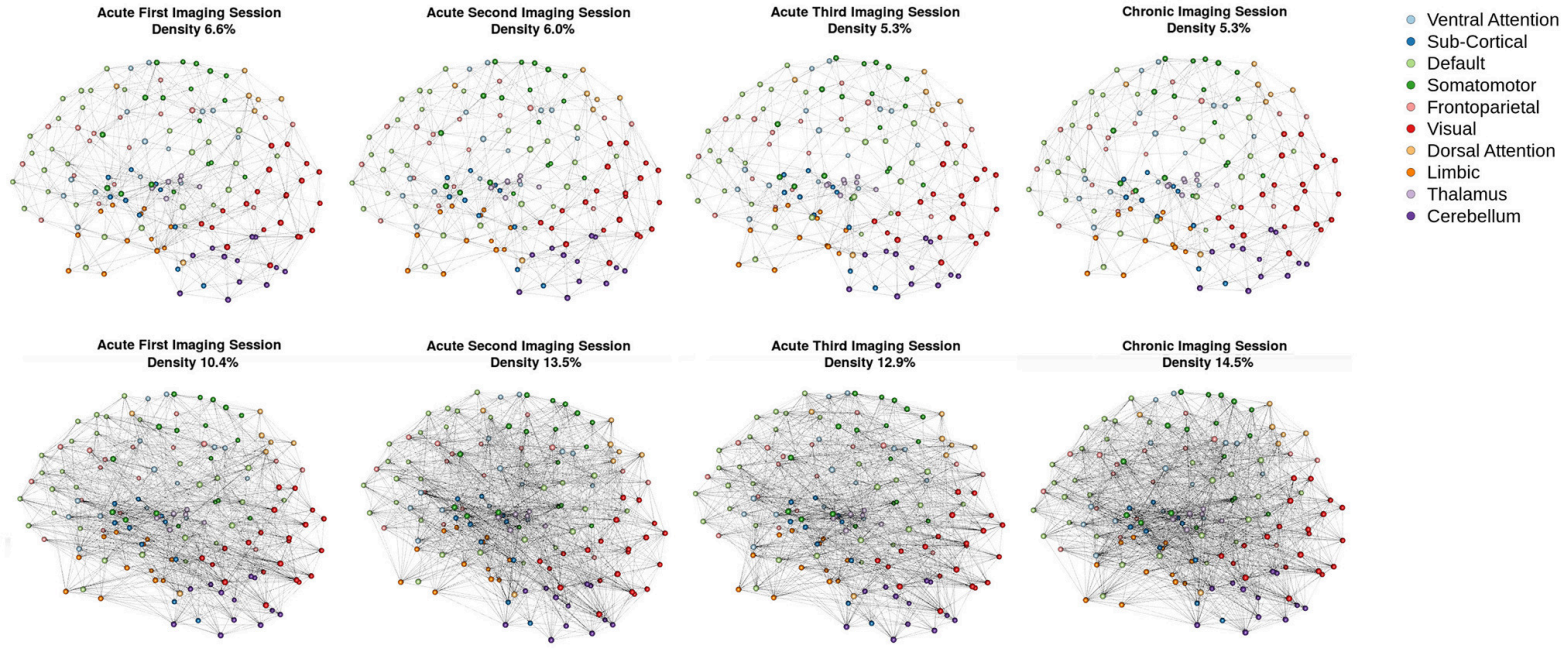
Patient recovery: network densities. Top Four Graphs are the thresholded [MANIA; (113)] structural connectivity. The first acute imaging session, second acute imaging session, third acute imaging session and chronic imaging sessions had 6.6, 6, 5.3, and 5.3% densities, respectively. Bottom Four Graphs are the thresholded functional connectivity using partial correlations [MoNeT; (116)]. The first acute imaging session, second acute imaging session, third acute imaging session and chronic imaging sessions had 10.4, 13.5, 12.9, and 14.5% densities, respectively.

Consistent with the argument we made in the introduction, as shown in Figure 3 (bottom row), the brain network construction using MoNeT resulted in four graphs with different estimated densities. Specifically, the three acute sessions returned graph densities of 10.4, 13.5, 12.9%, for the first, second, and third time-points, respectively, while the chronic session presented a graph density of 14.5%. Overall, then, the density differential between acute session 1 and chronic session was 4.1%, and the general acute-to-chronic pattern appeared to be a trend toward greater density. The structural connectivity (Figure 3, top row), on the other hand, had less variability in the densities of the graphs over time (i.e., 6.6, 6, 5.3, and 5.3%; a total difference of 1.3% between acute session 1 and chronic session).

#### 3.2.1. Integrating functional and structural connectivity

**Figure 4:**
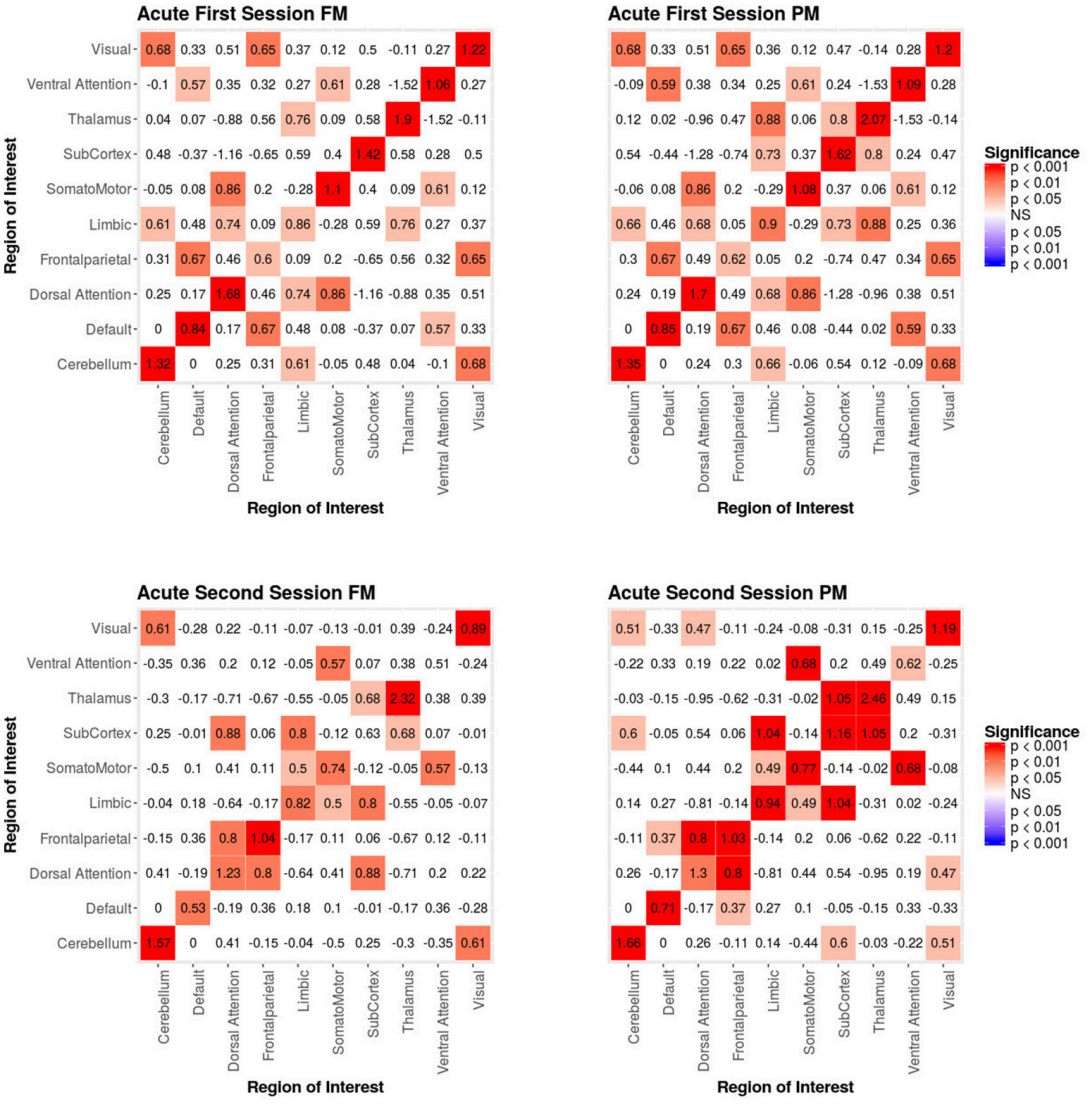
Patient recovery ERGM. Comparison of results for the FM and PM for acute sessions 1 and 2. The left figures display the FM mixing term results for the Acute first and second sessions. The mixing term term accounts for the inter- and intra-regional connectivity. The legend displays tints of red for significant positive parameter estimates. The right figures display the PM mixing term results for the Acute first and second sessions. The coloring scheme is the same as the FM. These figures are symmetric within each model because the graphs are undirected.

When we compared the properties of the network as estimated relying exclusively on functional connectivity (i.e., PM) as compared to when both functional and structural connectivity were jointly considered (i.e., FM), the PM included two significant positive inter-regional connectivity parameters (i.e., between thalamus and subcortex and between limbic network and subcortex; see top of Figure 4) which were no longer significant once structural connectivity was included (i.e., in the PM), suggesting their spurious status. More broadly, the positive parameter estimates became less positive and the negative parameter estimates became more negative. The only structural terms that were significant were the nodal covariate mixing term for connectivity between latent clusters 2 and 3 and within latent clusters 3 (see Table 2).

**Table 2:** Patient recovery ERGM.

	**ERGM Parameter Estimates**			
	**First Acute**		**Second Acute**	
	**PM**	**FM**	**PM**	**FM**
Edges	−6.29^***^	−6.34^***^	−7.64^***^	−7.71^***^
	(0.28)	(0.56)	(0.36)	(0.59)
Nodal Covariate: Degree (Structural)		0.00		0.00
		(0.00)		(0.01)
Nodal Covariate: Local Efficiency (Structural)		0.10		0.35
		(0.44)		(0.35)
Nodal Covariate: Cluster Coefficient (Structural)		−0.08		−0.33
		(0.34)		(0.29)
Nodal Covariate Mixing: Latent Cluster 1 to 1 (Structural)		0.03		1.01^***^
		(0.08)		(0.15)
Nodal Covariate Mixing: Latent Cluster 2 to 2 (Structural)		0.07		0.82^***^
		(0.17)		(0.11)
Nodal Covariate Mixing: Latent Cluster 1 to 3 (Structural)		−0.11		0.33^**^
		(0.08)		(0.12)
Nodal Covariate Mixing: Latent Cluster 2 to 3 (Structural)		−0.28^*^		0.16
		(0.12)		(0.11)
Nodal Covariate Mixing: Latent Cluster 3 to 3 (Structural)		0.24^*^		0.91^***^
		(0.10)		(0.12)
Nodal Covariate Mixing: Latent Cluster 1 to 4 (Structural)				0.23
				(0.13)
Nodal Covariate Mixing: Latent Cluster 2 to 4 (Structural)				0.22
				(0.12)
Nodal Covariate Mixing: Latent Cluster 3 to 4 (Structural)				−0.09
				(0.12)
Nodal Covariate Mixing: Latent Cluster 4 to 4 (Structural)				0.86^***^
				(0.13)
GWESP (Fixed 0.45)	2.09^***^	2.07^***^	3.11^***^	2.94^***^
	(0.13)	(0.13)	(0.21)	(0.20)

*Parameter estimates for the FM and PM of the Acute first and second sessions. The mixing term for resting state are excluded because they are in Figure 4. All of the structural parameter estimates are listed in the FM columns. The edges and GWESP parameter estimates are for the functional connectivity in the PMs and FMs. The LATEX code to create this table was produced by the R package called texreg (132)*.

* *p < 0.05*;

** *p < 0.01*;

*** *p < 0.001*.

At the second acute time-point, the PM and the FM again differed, with the latter showing an additional significant positive parameter estimate for connections between dorsal attention network and subcortex (see bottom Figure 4), three inter-regional connectivity parameter estimates that became non-significant (i.e., connections between cerebellum and subcortex, default network and frontoparietal network and visual network and dorsal attention; see bottom Figure 4) and two intra-regional connectivity parameter estimates that became non-significant (i.e., connections within the subcortex and ventral attention network; see bottom Figure 4). Overall, the parameter estimates both increased and decreased in magnitude with or without changing significance. Similar to the first acute session, the structural terms were only significant for the nodal covariate mixing term (i.e., between latent clusters 1 and 3, and within latent clusters 1, 2, 3, and 4; see Table 2).

**Figure 5:**
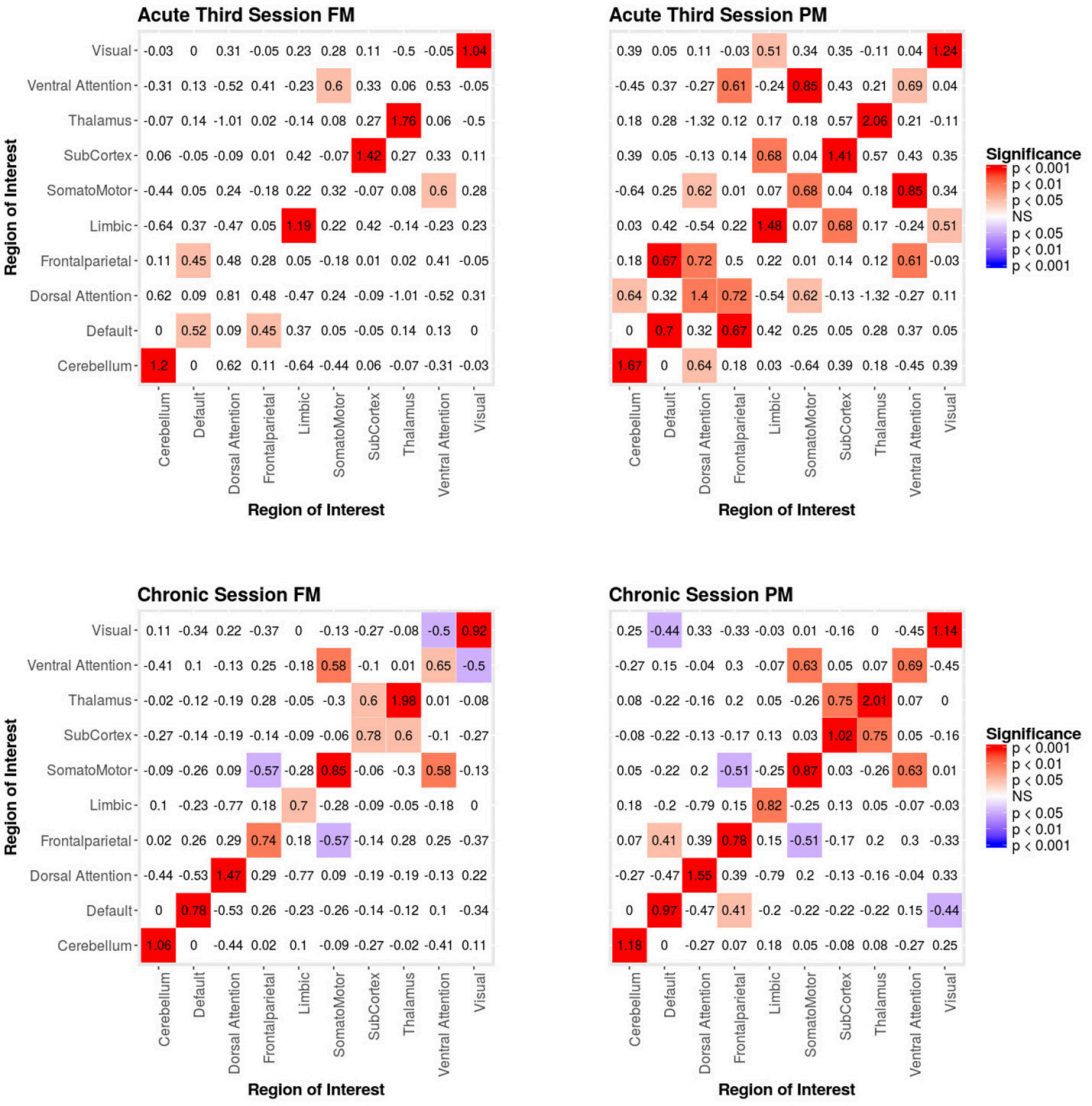
Patient recovery ERGM. Comparison of results for the FM and PM for acute session 3 and chronic session. The left figures display the FM mixing term results for the Acute third session and Chronic session. The mixing term term accounts for the inter- and intra-regional connectivity. The legend displays tints of red for significant positive parameter estimates and the significant negative parameter estimates are colored in tints of blue. The right figures display the PM mixing term results for the Acute third session and Chronic session. The coloring scheme is the same as the FM. These figures are symmetric within each model because the graphs are undirected.

In the third acute session, six inter-regional positive parameter estimates (i.e., connections between cerebellum and dorsal attention network, frontoparietal network and dorsal attention network, frontal parietal network and ventral attention network, dorsal attention network and somatomotor network, limbic network and visual network and limbic network and subcortex; see right Figure 5) and three intra-regional positive parameter estimates (i.e., connections within the dorsal attention network, somatomotor network and ventral attention network; see Figure 5) became non-significant once structural connectivity was included in the model. Similar to the first acute session, the parameter estimates generally decreased in magnitude. Finally, consistent with the first two acute sessions, the only significant structural feature was the nodal covariate mixing term (i.e., between latent clusters 2 and 3, latent clusters 1 and 4, latent clusters 1 and 6, latent clusters 3 and 6 and latent clusters 5 and 7, and within latent clusters 1, 2, 3, 4, 5, 6, and 7; see Table 3).

**Table 3:** Patient Recovery ERGM.

	**ERGM Parameter Estimates**			
	**Third Acute**		**Chronic**	
	**PM**	**FM**	**PM**	**FM**
Edges	−7.97^***^	−7.27^***^	−8.05^***^	−8.07^***^
	(0.36)	(0.63)	(0.42)	(0.57)
Nodal Covariate: Degree (Structural)		−0.01		0.01
		(0.01)		(0.01)
Nodal Covariate: Local Efficiency (Structural)		0.02		−0.11
		(0.12)		(0.16)
Nodal Covariate: Cluster Coefficient (Structural)		−0.22		0.33
		(0.15)		(0.17)
Nodal Covariate Mixing: Latent Cluster 1 to 1 (Structural)		2.33^***^		0.34
		(0.42)		(0.24)
Nodal Covariate Mixing: Latent Cluster 2 to 2 (Structural)		1.17^***^		−0.06
		(0.23)		(0.24)
Nodal Covariate Mixing: Latent Cluster 1 to 3 (Structural)		−0.48		−0.34^*^
		(0.44)		(0.17)
Nodal Covariate Mixing: Latent Cluster 2 to 3 (Structural)		0.47^*^		−0.51^**^
		(0.23)		(0.17)
Nodal Covariate Mixing: Latent Cluster 3 to 3 (Structural)		1.24^***^		0.29
		(0.24)		(0.15)
Nodal Covariate Mixing: Latent Cluster 1 to 4 (Structural)		1.25^***^		−0.52^**^
		(0.26)		(0.20)
Nodal Covariate Mixing: Latent Cluster 2 to 4 (Structural)		0.35		−0.55^**^
		(0.24)		(0.19)
Nodal Covariate Mixing: Latent Cluster 3 to 4 (Structural)		0.35		−0.55^***^
		(0.23)		(0.16)
Nodal Covariate Mixing: Latent Cluster 4 to 4 (Structural)		1.11^***^		0.56^**^
		(0.23)		(0.18)
Nodal Covariate Mixing: Latent Cluster 1 to 5 (Structural)		−0.35		−0.20
		(0.51)		(0.20)
Nodal Covariate Mixing: Latent Cluster 2 to 5 (Structural)		−0.01		−0.26
		(0.26)		(0.20)
Nodal Covariate Mixing: Latent Cluster 3 to 5 (Structural)		0.27		−0.52^**^
		(0.26)		(0.17)
Nodal Covariate Mixing: Latent Cluster 4 to 5 (Structural)		0.16		−0.39^*^
		(0.26)		(0.19)
Nodal Covariate Mixing: Latent Cluster 5 to 5 (Structural)		2.09^***^		0.42
		(0.31)		(0.23)
Nodal Covariate Mixing: Latent Cluster 1 to 6 (Structural)		1.20^***^		−0.42^*^
		(0.30)		(0.20)
Nodal Covariate Mixing: Latent Cluster 2 to 6 (Structural)		0.60^*^		−0.37^*^
		(0.24)		(0.18)
Nodal Covariate Mixing: Latent Cluster 3 to 6 (Structural)		−0.95^*^		−0.23
		(0.40)		(0.16)
Nodal Covariate Mixing: Latent Cluster 4 to 6 (Structural)		0.39		−0.22
		(0.24)		(0.17)
Nodal Covariate Mixing: Latent Cluster 5 to 6 (Structural)		0.37		−0.03
		(0.29)		(0.18)
Nodal Covariate Mixing: Latent Cluster 6 to 6 (Structural)		1.74^***^		0.30
		(0.29)		(0.19)
Nodal Covariate Mixing: Latent Cluster 1 to 7 (Structural)		−0.54		
		(0.51)		
Nodal Covariate Mixing: Latent Cluster 2 to 7 (Structural)		0.42		
		(0.24)		
Nodal Covariate Mixing: Latent Cluster 3 to 7 (Structural)		0.28		
		(0.25)		
Nodal Covariate Mixing: Latent Cluster 4 to 7 (Structural)		−0.15		
		(0.27)		
Nodal Covariate Mixing: Latent Cluster 5 to 7 (Structural)		0.59^*^		
		(0.26)		
Nodal Covariate Mixing: Latent Cluster 6 to 7 (Structural)		0.30		
		(0.27)		
Nodal Covariate Mixing: Latent Cluster 7 to 7 (Structural)		1.48^***^		
		(0.26)		
GWESP (Fixed 0.45)	3.23^***^	2.87^***^	3.48^***^	3.28^***^
	(0.20)	(0.20)	(0.24)	(0.24)

*Parameter estimates for the FM and PM of the Acute third session and Chronic session. The mixing term for resting state are excluded because they are in Figure 5. All of the structural parameter estimates are listed in the FM columns. The edges and GWESP parameter estimates are for the functional connectivity in the PMs and FMs. The LATEX code to create this table was produced by the R package called texreg (132)*.

* *p < 0.05*;

** *p < 0.01*;

*** *p < 0.001*.

In the chronic session, two inter-regional positive parameter estimates became non-significant after inclusion of the structural connectivity terms (i.e., between default network and frontoparetial network and default network and visual network; see right Figure 5). Conversely, unlike in the acute sessions, we also observed the reverse effect, with the the visual network and ventral attention network parameter estimate became significant in the FM. Additionally, the structural terms were only significant for the nodal covariate mixing term (i.e., between latent clusters 1 and 3, latent clusters 2 and 3, latent clusters 1 and 4, latent clusters 3 and 5, latent clusters 4 and 5, latent clusters 1 and 6 and latent clusters 2 and 6 and within latent clusters 4; see Table 3).

**Figure 6:**
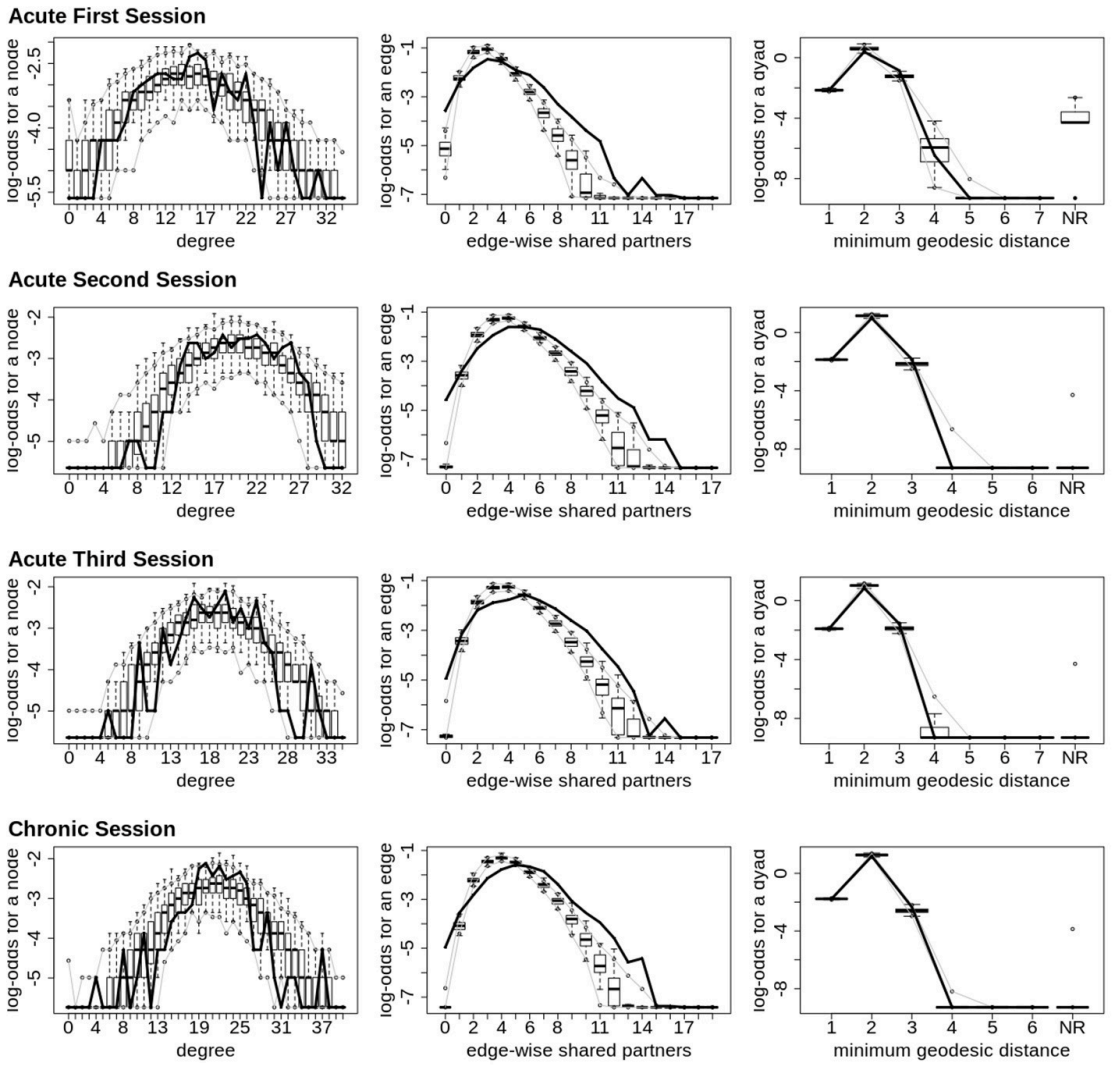
Patient recovery ERGM. Goodness of fit plots for the four FM (i.e., Acute Session 1, Acute Session 2, Acute Session 3 and Chronic Session). The black line marks the respective networks; the box-and-wiskers indicate the model data obtained from the 1000 simulations of each model (see section 2.6).

Finally, across all imaging sessions the GWESP parameter estimate was reduced in magnitude (see Tables 2, 3) by the addition of the structural terms, with the largest difference seen in third acute session (see Table 3). Additionally, the GOF (see Figure 6) are fit for every statistic in all of the FM. All the GOF terms fit well except for a portion of the edge shared partners, but in the model statistics (the far right in Figure 6) are well fit to the original data.

As we will discuss below, the differences we are reporting between the results obtained with the conventional model (i.e., PM), estimated form functional connectivity alone, and those obtained with the (i.e., FM), estimated from both the functional and structural connectivity, demonstrates the risk of drawing spurious conclusions when relying on the PM.

### 3.3. STERGM

The STERGM allowed us to look at the temporal dynamics of recovery post severe brain injury with two parallel models: a formation model and a dissolution model. The formation model produces parameter estimates describing how likely it is that new connections (i.e., edges) form throughout the recovery from coma, while the dissolution model produces parameter estimates describing how likely it is that existing connections dissolve (or persist) throughout recovery.

**Figure 7:**
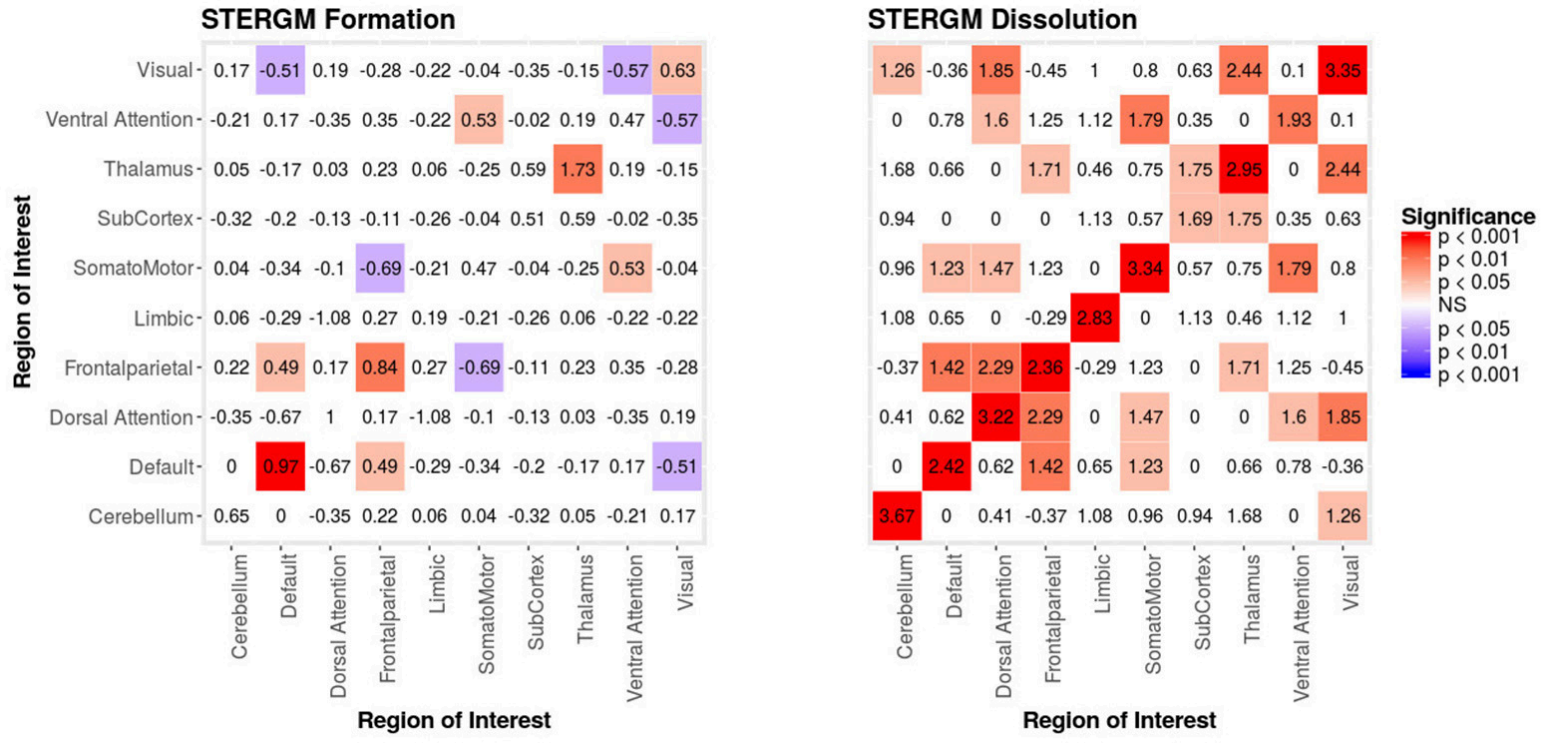
Patient Recovery STERGM. Results for the formation (**left**) and dissolution (**right**) models over 6 months. The mixing term accounts for the inter- and intra-regional connectivity that form over 6 months. The legend displays tints of red for significant positive parameter estimates and the significant negative parameter estimates are colored in tints of blue. The right figure displays the dissolution model STERGM mixing term results. The coloring scheme is the same as the formation model, but the mixing term represents the connectivity that are dissolved or preserved over 6 months. These figures are symmetric within each model because the graphs are undirected.

**Figure 8:**
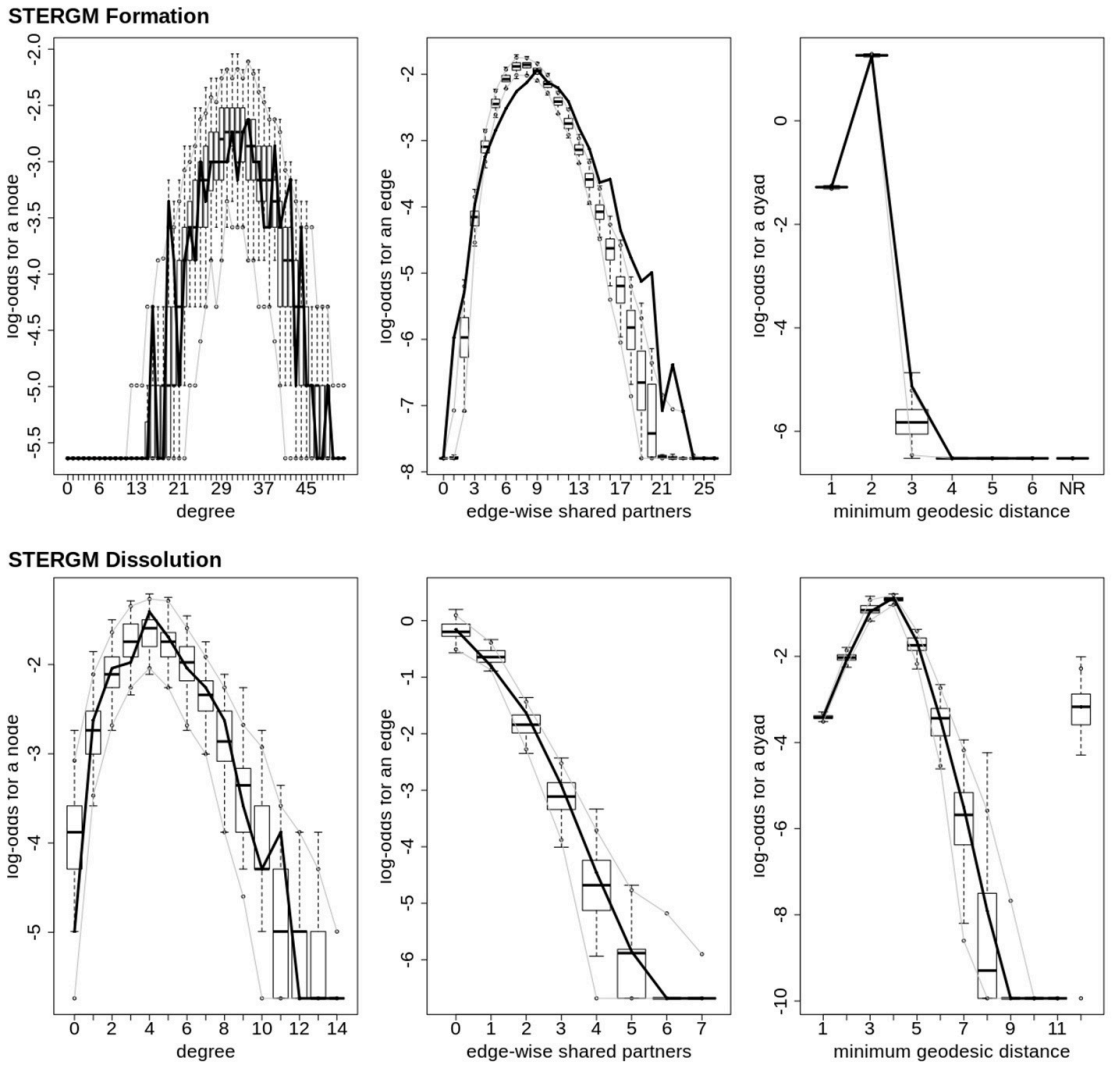
Patient recovery STERGM. Goodness of fit plots for the formation (**top**) and dissolution (**bottom**) models. The black line marks the formation and dissolution networks observed over time in the patient's graphs between the first Acute session and the Chronic session; the box-and-wiskers indicate the model data obtained from the 1000 simulations of each model (see section 2.6).

In our index patient, the formation model showed a significant negative edges parameter estimate and a significant positive GWESP parameter estimate, the latter implying a tendency to form edges over time that close triangles (see Table 4). Additionally, none of the structural nodal covariates were found to be significant (see Table 4). There were, however, four significantly positive parameter estimates for intra-regional connectivity (i.e., default network, frontoparietal network, thalamus, and visual network; see left Figure 7), three significantly negative parameter estimates for inter-regional connectivity (i.e., between default network and visual network, somatomotor network and frontoparietal network, and ventral attention network and visual network; see left Figure 7), and two significantly positive parameter estimates for inter-regional connectivity (i.e., between default network and thalamus, and somatomotor network and ventral attention network; see left Figure 7). The dissolution model has a significantly negative edges parameter estimate and significantly positive GWESP parameter estimate (see Table 4). Also, none of the structural terms were significant for the dissolution model. Additionally, all ten parameter estimates for intra-regional connectivity (i.e., cerebellum, default network, dorsal attention network, frontoparietal network, limbic network, somatomotor network, subcortex, thalamus, ventral attention network, and visual network) significantly positive (see right Figure 7) and 11 significantly positive parameter estimates for inter-regional connectivity (i.e., between cerebellum and visual network, default network and frontoparietal network, dorsal attention network and frontoparietal network, dorsal attention network and somatomotor network, dorsal attention network and ventral attention network, dorsal attention network and visual network, frontoparietal network and thalamus, somatomotor network and ventral attention network, subcortex and thalamus, and thalamus and visual network; see right Figure 7). Finally, the GOF (see Figure 8) were fit well for every statistic in both the formation and dissolution model. Overall, the model was thus well fit for both the formation and dissolution models. All the GOF terms fit well except for a portion of the edge shared partners, but in the model statistics are well fit to the original data.

**Table 4:** Patient Recovery STERGM.

	**STERGM Parameter Estimates**	
	**Formation**	**Dissolution**
Edges	−10.03^***^	−3.56^*^
	(1.04)	(1.79)
Nodal Covariate: Degree (Structural)	0.01	0.03
	(0.01)	(0.02)
Nodal Covariate: Local Efficiency (Structural)	−0.14	−1.27
	(0.64)	(1.64)
Nodal Covariate: Cluster Coefficient (Structural)	0.34	1.33
	(0.49)	(1.25)
Nodal Covariate Mixing: Latent Cluster 1 to 1 (Structural)	−0.04	−0.01
	(0.09)	(0.21)
Nodal Covariate Mixing: Latent Cluster 2 to 2 (Structural)	0.04	0.30
	(0.17)	(0.41)
Nodal Covariate Mixing: Latent Cluster 1 to 3 (Structural)	−0.12	−0.11
	(0.09)	(0.24)
Nodal Covariate Mixing: Latent Cluster 2 to 3 (Structural)	−0.04	0.19
	(0.11)	(0.32)
Nodal Covariate Mixing: Latent Cluster 3 to 3 (Structural)	−0.00	−0.13
	(0.14)	(0.32)
GWESP (Fixed 0.75)	3.26^***^	
	(0.33)	
GWESP (Fixed 0.25)		0.27^***^
		(0.08)

*Parameter estimates for the formation and dissolution models. The mixing term for resting state are excluded because they are in Figure 7. All of the structural parameter estimates are listed in the FM columns. The edges and GWESP parameter estimates are for the functional connectivity in the formation and dissolution models. The LATEX code to create this table was produced by the R package called texreg (132)*.

* *p < 0.05*;

^**^p < 0.01;

*** *p < 0.001*.

## 4. Discussion

In this work, we have addressed four issues which, while general to the implementation of network theory in the field of functional neuroimaging, are particularly relevant to studies in the clinical context of DOC. In what follows we discuss how the approach we have demonstrated above in a patient recovering from coma resolves specifically each of the four problems outlined in the introduction. The first three problems discussed were solved using a single model which controls for (i) the density of the functional connectivity, (ii) the effects of variance/change in structural connectivity on the functional metrics, while (iii) modeling the intra- and inter-connectivity of the resting state networks and the effects of higher order terms (i.e., GWESP). The final problem was resolved using STERGM to model the network dynamics in recovering from coma.

### 4.1. Solution to problem #1: use natural density, not arbitrarily fixed density (i.e., use a multiple regression framework—part I)

As our longitudinal data shows, consistent with results from other domains of neuroscience [see (45, 133)], brain graphs are susceptible to having different “natural” levels of density at which they are the most stable and which might thus be ideal to estimate network properties. In our data, over the progression of 6 months post injury, as the patient recovered consciousness and cognitive function, the natural brain graph density went from 10.4 to 14.5%. These density differences were revealed thanks to the use of MoNeT (116), a tool which combines a penalized maximum likelihood estimation with a resampling-based (bootstrapped) model selection procedure in order to find the most stable level of sparse brain graph given a set of time-dependent measurements (e.g., fMRI data). On the one hand, as we will explain below, these differences might well reflect important aspects of network dynamics in the recovery of consciousness post severe brain injury. On the other hand, regardless of the ultimate interpretation of the finding in of itself, had we employed the standard approach and enforced equal density across brain graphs in order to allow comparability (42, 55), these differences would have been obscured and would have introduced a bias in the direct comparison of topological properties across graphs. Ultimately, an accurate estimation of the connectivity is necessary to correctly model the connectivity. ERGM and STERGM allow for controlling the density without having to fix the density for all graphs. This allows for data driven approaches to allow the density to vary based on the stability of the connectivity estimates. This natural variance could reveal differences in graph statistics that would otherwise be masked by fixing density. Overall, this result further demonstrates that, when arbitrarily enforcing equal density across graphs, we are in fact biasing our results toward the graphs with natural density closest to the threshold employed. While we show this in the context of time, it immediately translates to cross-sectional analyses that are also typical of the field of DOC (e.g., healthy controls vs. patients), with the prediction that the more different the natural density across groups, the greater the bias in the results.

### 4.2. Solution to problem #2: control for interrelations across network metrics (i.e., use a multiple regression framework—part II)

As discussed above, ERGM can cope with comparing graphs with different natural densities because it factors in density as a variable in the model (in other words, it controls explicitly for different densities). Similarly, ERGM can also control for interrelations across the many metrics that are typically estimated by explicitly including them all in a single model. As mentioned in the introduction, this approach is akin to performing a multiple regression model in which each network feature is evaluated for its unique contribution to the graph, as opposed to the current graph theoretic approach dominating in neuroimaging, which is akin to running several single-variable regressions, one per topological feature investigated. The Florentine business networks were used to demonstrate the effect of leaving out significant contributing factors to the model, something that renders our ERGM vulnerable to correlations between graph properties similar to the current conventional approached (42). As shown in Table 1, using PMs can lead to incorrectly estimating the magnitude or the significance of network measures. For example, in network A (Figure 1, left), the failure to include the mixing terms leads to a significant GWESP term, however, it appears to be overestimated as compared to the FM (where it is not significant). In other words, on the basis of the PM results, one would be justified in concluding that triadic closure (i.e., the tendency for edges to appear where they complete triangles) is a key stochastic process underlying the network. Yet, the FM shows that this result is spurious and is in fact due to the mixing term— that is, to the dynamics of within-group connectivity, and not triadic closure. As shown in Table 1, changing group membership of one node alone, preserving all other aspects of the network, affected both qualitatively and quantitatively the network measures (compare the FM columns for PM_*A*_ and PM_*B*_ in Table 1). Similarly to Network A, Network B's PMs returned different parameter estimates than the FM. As we will discuss below, a similar effect is at play in the neuroimaging data where, failure to include structural information, could have lead to incorrectly attributing to functional connectivity between the fronto-parietal and the default mode networks a network characteristic that is in fact due to structural connectivity (i.e., problem #3, cf., Figure 4, 5).

### 4.3. Solution to problem #3: adjust for the effects of structural connectivity on functional connectivity (i.e., use a multiple regression framework—part III)

As shown in the results, ERGM is capable of addressing the currently unresolved issue of integrating functional and structural connectivity in a unique framework (37, 38, 40). Analogously to the two previous points, the solution employed by ERGM is to include structural connectivity terms in the model, thus explicitly adjusting for the relationship between the structural and functional connectivity. In our data, inclusion of structural terms in the model affected all other parameter estimates, empirically demonstrating that, in the context of recovery of consciousness after severe brain injury, failing to include structural connectivity is tantamount to mis-specifying the model [similarly to not including network density (i.e., problem #1)] or not modeling all estimated metrics in a single model [(i.e., problem #2)]. While we recognize that this is likely to be an issue in any field where structural connectivity might differ across groups and/or individuals, there is also little doubt that this is particularly problematic in the context of DOC where the underlying structural architecture is likely to be substantially different from healthy volunteers [e.g., (64, 134)], across different clinical groups [e.g., (69)], and over time [e.g., (96, 135) as well as in the data presented here].

Specifically, our results show that when structural data are included (i.e., in the FMs), the probability of inter- and intra-regional connectivity changes—as compared to the PMs—including: parameter estimates with a higher magnitude in the PM (e.g., connections between default network and ventral attention network, limbic network to thalamus, and within limbic network in the Acute First session), parameters with a lower magnitude in PM (e.g., connections between visual network and cerebellum, visual network and subcortex or visual network and thalamus in the Acute Second session), and parameters which went from non-significant in the PM to significant in the FM (e.g., connections between dorsal attention network and subcortex in the Acute Second session or connections between visual network and ventral attention in the Chronic session) and viceversa (e.g., connections between default network and frontoparietal network in the Chronic session or connections between thalamus and subcortex in the Acute First session). These results have immediate theoretical implications for the field of DOC in as much as the partial ERGM model in our patient shows increased likelihood of connectivity between the default mode and the fronto-parietal networks throughout recovery from coma (see Figure 4, 5). This could be (mistakenly) construed as bearing on the issue of the relationship between the “external awareness” and “internal awareness” networks in DOC (136, 137). For example, the relationship between these two networks was no longer observed once structural data was included in the FM exposing the initial finding as spurious and likely reflecting improper attribution of variance due to leaving out the structural terms from the model.

Finally, we note that ERGM has an important advantage over other techniques in the context of integrating functional and structural connectivity. Indeed, previous approaches only made use of the structural connectivity in order to predict the functional network (71, 72) or in order to jointly estimate the functional and structural connectivity (74–76). ERGM, however, allows estimating the influence of structural connectivity on the properties of the functional networks, something which, even at the level of one patient alone, has a large enough effect to change the significance and/or magnitude of the network's parameter estimates.

### 4.4. Solution to problem #4: assess dynamics of change across time-points, not static differences across time-points

Finally, an additional advantage of this new approach is the ability to directly analyze network dynamics over time—an issue that is very important in the context of loss and recovery of consciousness after severe brain injury (28, 34). In our example data, the two STERGM models uncovered a strong positive parameter estimates for intra-regional connectivity in all networks, for the dissolution model, indicating that in the process of recovery there are strong tendencies to preserve existing edges across time. Additionally, there are four positive parameter estimates for the formation of new edges, implying that as our patient recovered he was more likely to establish new connectivity within and between networks. Taken together, the tendency of our patient to maintain existing connections and develop novel ones might well explain why we observed a tendency over time for the “natural” density of networks to increase throughout recovery. It should also be pointed out that while we did not find any negative parameter estimate in the dissolution model, a significant negative estimate could be interpreted as evidence for neural reorganization, another important advantage of ERGM in the context of DOC [e.g., (95)].

### 4.5. Limitations

It is important to consider two important limitations of the work above. First, we have presented the use of ERGM in the context of a single patient. On the one hand, ERGM was specifically developed to allow meaningful analysis of single graphs. Indeed, unlike neurosciences and other experimental biological and behavioral sciences, some fields do not typically have multiple graphs to compare (e.g., multiple subjects, multiple time-points), but rather have a single graph from which meaningful inferences are drawn [e.g., sociology; (2), transportation (6), and public health (4)]. On the other hand, although—formally—inferences could be legitimately drawn from a single case, in the context of DoC and clinical work, brain-derived network analyses reflect much of the heterogeneity of the underlying conditions, thus making inferences drawn from individual cases questionable in their generality and applicability to other patients. Furthermore, at this initial stage, there are no baseline or control measurements against which to compare one patients' parameters derived from the (ST)ERGM. Second, because of the pragmatics and reality of clinical work, acute scans, which happened in an in-patient setting, were performed on a 3 Tesla Siemens TimTrio system while follow-up MR data were acquired in an out-patient setting, on a 3 Tesla Siemens Prisma system. The impact of such a variable on the model parameters remains to be assessed in larger samples, including in healthy volunteers. We thus leave it to future cohort studies to interpret in detail the significance of the specific ERGM and STERGM parameters with respect to the issue of loss and recovery of consciousness after severe brain injury.

## 5. Conclusions and future work

Network analyses are an attempt to synthesize complex processes into a small number of metrics. In this paper we have introduced a novel [in the context of DOC, for other contexts within neuroimaging, cf.: (89–91)] approach to estimating network properties, ERGMs, which overcome four important challenges faced by current graph theoretic approaches to brain data and which are particularly consequential in the context of DOC. The main advantage of ERGM over current approaches is the fact that it adopts a multiple regression framework *in lieu* of multiple parallel simple regressions (i.e., one per each metric). Under this multiple regression framework, brain networks can be compared across densities—since the density of each will be controlled for within the model. This side-steps the issue of having to impose the same arbitrary sparsity across networks which are likely to have very different stable levels of density, as is the case, for example, between severely brain injured patients and controls or in longitudinal recovery. Similarly, by including in a unified model structural and functional data, it is possible to acknowledge and control for the fact that patients surviving severe brain injury are likely to have very heterogeneous brain pathology and thus profound differences in structural substrate—a fact that is currently ignored in the extant literature. Even in one patient alone, direct comparison of the conventional PM with the FM demonstrated how failing to consider structural information can lead to spurious results and erroneous conclusions. Furthermore, ERGM can be extended to assess dynamics of change thus allowing to discover the network evolution that govern loss and recovery of consciousness over time, as opposed to comparing static graphs at different time-points.

Finally, we end this paper by pointing out that the reader can implement (ST)ERGM as performed here using the freely distributed ergm package (40) in R and the Markov Network Toolbox [MoNeT; (116)] in MATLAB.

## Author contributions

JD, MM, and PV designed the experiment. JD and MJ analyzed the data. JD and MM interpreted the results. JD drafted the manuscript, all authors provided feedback.

### Conflict of interest statement

The authors declare that the research was conducted in the absence of any commercial or financial relationships that could be construed as a potential conflict of interest.
